# Editorial Comment: External validation of nomogram to predict inguinal lymph node metastasis in patients with penile cancer and clinically negative lymph nodes

**DOI:** 10.1590/S1677-5538.IBJU.2018.0756.1

**Published:** 2019-09-02

**Authors:** Luciano A. Favorito

**Affiliations:** 1 Professor Titular, Unidade Urogenital da Univ. Est. do Rio de Janeiro – UERJ, RJ, Brasil;; 2 Urologista do Hospital da Lagoa Federal, Rio de Janeiro , RJ, Brasil;; 3 Editor Associado do International Braz J Urol Brasil

In this interesting paper Dr. Maciel and collegues from Sao Paulo – Brazil conduct an external validation of a previously developed nomogram (1) to predict inguinal lymph node (ILN) metastases in penile cancer in patients with clinically negative lymph nodes. The authors analyzed 65 men with penile cancer who underwent inguinal lymph node dissection. Of 65 men, only 24 (36.9%) presented with positive LNs. The authors concluded that the present nomogram applied in Brazilian population had low accuracy and low precision for correctly identifying patients with penile cancer who have positive ILN.

Penile cancer is a rare neoplasia with low incidence in developed countries. In Brazil the incidence rate of penile cancer is 2.9 - 6.8/100,000 inhabitants, resulting in this country having one of the world’s highest incidence rates for this neoplasia (2-4). The most common sites of penile cancer metastasis are the superficial and deeper nodes of the inguinal and iliac region. The occurrence and extent of inguinal lymphatic metastasis are the most important prognostic factors in patients with penile cancer and usually imply worse oncologic prognosis (5). Extended Inguinal lymphadenectomy (open, laparoscopic or robotic) is the most useful and commonly performed surgery for staging and to cure inguinal metastasis in penile cancer cases. Although it is a widespread technique, post operatory complications often occur (6-8).

This paper is very important, but in the future, papers with prospective studies and with a more significant sample will be necessary to confirm the application of this nomogram to predict inguinal lymphatic metastasis in patients with penile cancer.
